# Rare Condition of Intrathoracic Phrenic Nerve Schwannoma Successfully Treated with Uniportal Video-Assisted Thoracoscopic Surgery

**DOI:** 10.1155/2021/3276843

**Published:** 2021-08-09

**Authors:** Lu Huu Pham, Kinh Quoc Nguyen, Hung Quoc Doan, Lanh Sy Nguyen, Ha Thi-Ngoc Doan

**Affiliations:** ^1^Center of Cardiovascular and Thoracic Surgery, Viet Duc University Hospital, Hanoi, Vietnam; ^2^Department of Surgery, Hanoi Medical University, Hanoi, Vietnam; ^3^Center of Anesthesia and Surgical Intensive Care, Viet Duc University Hospital, Hanoi, Vietnam; ^4^Department of Pathology, Viet Duc University Hospital, Hanoi, Vietnam; ^5^Hanoi Medical University, Vietnam

## Abstract

**Introduction:**

Neurogenic tumors in the mediastinum account for approximately 20-30% of all types of mediastinal tumors in adults. This pathology is usually benign and has no or very few symptoms. Schwannoma rarely involves the phrenic nerve. We report a unique case of schwannoma involvement of phrenic nerve. *Case Presentation*. The 43-year-old female patient has an annual check-up of computerized tomography to detect the mass in the right middle mediastinum, so the patient was admitted to the hospital. Chest computerized tomography image found a mass of the middle mediastinum with the size of 23 × 22.3 mm located between the right pulmonary artery and the pericardium with uniform margins and clear boundaries, not invading the surrounding organization. Very little contrast is absorbed after injection. She underwent a uniportal video-assisted thoracoscopic surgery, and this mass was found to be originating from the right phrenic nerve. Resection of the portion of phrenic nerve with mass was performed. Postoperatively, the patient was discharged from the hospital after 4 days of treatment in a clinical condition with no difficulty breathing and no chest pain; postoperative X-ray showed no abnormality, and the right diaphragm was unchanged.

**Conclusion:**

Although they are very rare, schwannomas of the phrenic nerve should be considered in the differential diagnosis of mediastinal tumors. Uniportal video-assisted thoracoscopic surgery is a preeminent option with properly sized tumors that deliver good results and have no postoperative complications associated with surgery.

## 1. Introduction

Neurogenic tumors account for approximately 20%-30% of all mediastinal tumors in adults [1–5]. They can originate in any neurogenic structures in the mediastinum, including the sympathetic or parasympathetic chain, intercostal nerves, and spinal ganglia. Most of these tumors are benign and with few or even no symptoms; thus, they are often detected by radiography or CT scanner incidentally [1, 6, 7]. Of these, schwannomas, the tumors sheathed with Schwan cells, and in phrenic nerve are very rare lesions that have sporadically been reported in few individual cases of literature [2, 5, 7, 8]. We reported a case of asymptomatic schwannomas in the middle mediastinum originating from the right phrenic nerve. The tumor was removed by uniportal video-assisted thoracoscopic surgery (uniportal VATS), a new minimally invasive technique, in a single surgical center with good postoperative results.

## 2. Case Presentation

**Figure 1:**
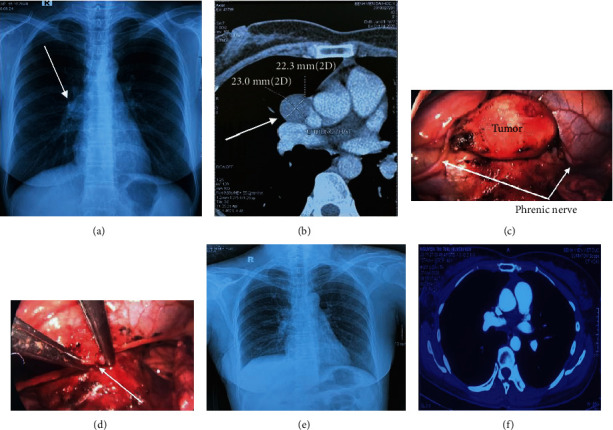
Preoperative, intraoperative, and postoperative images: (a) preoperative chest X-ray (arrow); (b) the middle mediastinal tumor (arrow); (c) intraoperative tumor and the right phrenic nerve (arrow); (d) removed tumor image (arrow); (e) postoperative chest X-ray; (f) the chest CT scanner 1 month after surgery.

The 43-year-old female patient has no special history, and she has an annual check-up of computerized tomography to detect the mass in the right middle mediastinum, so the patient is admitted to the hospital. On-board examination, the patient was awake with good overall condition and has no abnormalities of the cardiovascular system and thoracic system during clinical examination. The manifestation on thoracic X-ray: right pulmonary hilum has an even, well-defined border (Figure 1(a)). Chest computerized tomography image showed a mass in the middle mediastinum with the size of 23 × 22.3 mm located between the right pulmonary artery and the pericardium with uniform margins and clear boundaries and did not invade the surrounding organization. A modest amount of contrast was absorbed after injection (Figure 1(b)). Echocardiography and respiratory function measurements were normal. Biochemical blood tests and complete blood count were in normal range. The preoperative diagnosis indicated that the tumor is located in the middle mediastinum with suspected mediastinal lymphadenopathy. The patient underwent uniportal video-assisted thoracoscopic surgery for tumor resection.

**Figure 2:**
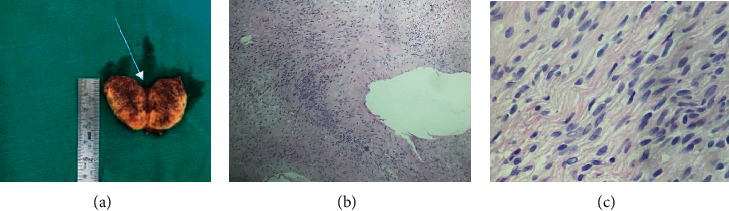
Images of postoperative pathologies: (a) gross picture; (b) HE × 50 and HE × 400, tumors with a dense cellularity and a small cystic degeneration areas; tumor cells were spindle-shaped and quite uniform and have fine chromatine, unknown nucleoli, no abnormalities, and poor mitoses. (c) The tumor cells have an unknown cytoplasmic boundary, arranged in parallel bands, suggesting a palisading.

*Operative procedure*. We made a 3 cm skin incision on the VI intercostal space in the right anteriolateral side. The intraoperative examination found that the tumor of the right diaphragmatic nerve was about 2 × 3 cm in size (Figure 1(c)) with clear tumor boundary to surrounding organs (including pericardium, pulmonary artery, and lung parenchyma), proving the noninvasiveness. We conducted a segmentation of the right phrenic nerve to remove the tumor and release the length of the phrenic nerve associated with reconnecting the right phrenic nerve with a prolene 8/0 through endoscopy (Figure 1(d)). The removed specimen was pale yellow with superficial hemorrhage (Figure 2). Postoperatively, the patient was discharged after 4 days of treatment with a clinical condition of no difficulty breathing and no chest pain, and postoperative X-ray showed no abnormality; the right diaphragm was unchanged (Figure 1(e)). CT scanner result 1 month after discharge showed no abnormal images compared to the preoperative results (Figure 1(f)).

## 3. Discussion

Intrathoracic neurogenic tumors are usually localized at posterior mediastinum. They could arise either from the intercostal nerves or spinal nerve roots [1, 5, 7]. However, they may rarely arise from the nerve sheaths that are located in the intrabronchial, parenchymal, and middle or anterior mediastinal region [5]. The origination from the phrenic nerve is very rare. Tanaka et al. [9] in a review of 138 mediastinal tumors found only one originated from the phrenic nerve. Most intrathoracic phrenic nerve schwannomas are reported to be asymptomatic (92% to 94%) [1]. On the other hand, several authors have reported clinical cases with some specific symptoms. Gilani and Danforth described a very rare schwannoma of left phrenic nerve with chronic intractable hiccups [4]. Surgery in this situation is usually required to improve the symptoms and quality of life of the patients. Several months after surgery, the hiccups of this patient were markedly reduced with no postoperative complications [4]. Orki et al. described a 43-year-old female patient that had left-sided chest pain for one month [5]. Moinuddeen et al. described a phrenic nerve schwannoma with diaphragmatic eventration and devastation of the lower lobe in the left [8].

Since there are almost no clinical symptoms, preoperative diagnosis is difficult. However, with a well-circumscribed tumor, with spherical or oval contours, located in the anterior or middle mediastinum, lateralized to one side, in contact with the pericardia, pulmonary artery, or superior vena cava, as in our patient, it was suspected to involve the phrenic nerve. Therefore, it was a diagnosis of exclusion, because thymoma, lymphoma, and teratoma are the most common tumors of the anterior mediastinum or hilum.

Surgical treatment of the tumor is the first and foremost option. Depending on the size of the tumor, either classical surgery or thoracoscopic surgery would be preferable.

The tumor could be easily resected through uniportal VATS in our case. Postoperative period showed no complications with normal diaphragm movement, and the patient was discharged at the 4^th^ postoperative day and reexamined 1 month after surgery. Most of authors agreed that patients with adequate pulmonary function preoperatively would remain asymptomatic after unilateral phrenic nerve resection. Otherwise, patients with poor pulmonary function or demonstrate postoperative symptoms from eventration after phrenic nerve resection may solve a diaphragmatic plication [1, 4]. Uniportal VATS has many proven advantages such as reduced surgical trauma, decreased postoperative pain, faster rehabilitation, and improved patient satisfaction compared with the conventional VATS [10]. However, the size and relation with the intrathoracic other organs should be considered to indication of this method.

Postoperative pathological results with our patient (as Figure 2) with HE × 50 and HE × 400 showed that tumor has a dense cellularity and a small cystic degeneration areas (Figure 2(b)), and tumor cells were spindle-shaped and quite uniform and have fine chromatine, unknown nucleoli, no abnormalities, and poor mitoses. The tumor cells have an unknown cytoplasmic boundary, arranged in parallel bands, suggesting a palisading (Figure 2(c)).

Neurogenic tumors in the mediastinum usually have the benign pathological results. However, some specific cases have malignant pathological results. Smahi et al. [3] reported malignant degeneration of neurofibromas which was greater in the context of von Recklinghausen disease and occurs in 10% of cases. Rais et al. [11] have published a case with the malignant pathological result of a phrenic nerve tumor that was managed successfully by multimodal treatment.

## 4. Conclusion

Intrathoracic phrenic nerve schwannomas are usually benign and slowly expanding and have very little clinical symptoms and almost no malignant transformation. Therefore, it is necessary to think about it and perform differential diagnosis with some types of tumors in the anterior and middle mediastinum, but they should be treated by surgical excision. Uniportal video-assisted thoracoscopic surgery is a preeminent option with properly sized tumors that deliver good results and have no postoperative complications associated with surgery.

## Data Availability

The raw data used to support the findings of this study are available from the corresponding author upon request.
